# Supplementary material for: Computationally driven discovery of SARS-CoV-2 Mpro inhibitors: from design to experimental validation
Source: Chem Sci. 2022 Feb 10;13(13):3674–87. doi: 10.1039/d1sc05892d (PMC8966641; doi:10.1039/d1sc05892d)
Supplement: SC-013-D1SC05892D-s001 [file SC-013-D1SC05892D-s001.pdf]

Electronic Supplementary Material (ESI) for Chemical Science.
This journal is © The Royal Society of Chemistry 2022

Computationally driven discovery of targeting SARS-CoV-2 Mpro inhibitors: from design to experimental validation†

Léa El Khoury,[a‡] Zhifeng Jing,[a‡] Alberto Cuzzolin,[b] Alessandro Deplano,[c] Daniele Loco,[a] Boris Sattarov,[a] Florent Hédin,[a] Sebastian Wendeborn,[d] Chris Ho,[a] Dina El Ahdab,[o] Theo Jaffrelot Inizan,[o] Mattia Sturlese,[g] Alice Sosic,[e] Martina Volpiana,[e] Angela Lugato,[e] Marco Barone,[e] Barbara Gatto,[e] Maria Ludovica Macchia,[e] Massimo Bellanda ,[f] Roberto Battistutta,[f] Cristiano Salata,[h] Ivan Kondratov,[i] Rustam Iminov,[i] Andrii Khairulin,[i] Yaroslav Mykhalonok,[i] Anton Pochepko,[i] Volodymyr Chashka-Ratushnyi,[i] Iaroslava Kos,[i] Stefano Moro,[g] Matthieu Montes,[l] Pengyu Ren,[m] Jay W. Ponder,[n] Louis Lagardère,[o] Jean-Philip Piquemal*[o,p] and Davide Sabbadin*[a,d]

Received Date
Accepted Date

DOI: 00.0000/xxxxxxxxxx

Supplementary Information

1 Equilibration protocol prior to MD simulations

Selected binding poses from the molecule docking were used for MD simulations. The protein-ligand complexes were solvated in a cubic water box with a minimum distance between protein and box edge of 12 Å. NaCl salt was added to neutralize the system and give a concentration of 0.15 mol/L.

The systems were equilibrated through multiple steps: (1) energy minimization to a threshold of 10 kcal/mol/$Å^2$; (2) 0.1 ns NVT simulations at 200 K, 2.0 fs time step, with harmonic restraints (force constant 1.0 kcal/mol/$Å^2$) on protein, ligand and crystallographic water; (3) 0.1 ns NVT simulations at 298 K, 2.0 fs time step, with harmonic restraints (force constant 1.0 kcal/mol/$Å^2$) on protein and crystallographic water, and flat-bottom restraint (force constant 1.0 kcal/mol/$Å^2$, radius 3.0 Å) on the ligand. (4) 1 ns NPT simulations at 298 K and 1 bar, with same restraints as last step; (5) 6 ns NPT simulations at 298 K and 1 bar, with harmonic restraints (force constant 1.0 kcal/mol/$Å^2$) on protein backbone.

Two independent equilibration simulations were conducted for each binding pose. The last 4 ns simulations for all binding poses of each ligand were used for clustering analysis. The ligand and heavy atoms of residues 41, 144, 145, 146, 163, 164 were used to calculate the RMSD. The DBSCAN algorithm with a cutoff distance of 0.8 Å and the hierarchical clustering algorithm with 1.1 Å cutoff for the average distance were used to select the largest clusters.

2 SMILES of the studied compounds

Compound	Smiles
QUB-00006	CSc1ccc2c(c1)NCC(F)(F)[C@@H]2C
QUB-00006-Int-07	CSc1ccc2c(c1)NC(=O)C(F)(F)C2=O
QUB-00006-Int-01	CSc1ccc2c(c1)NC(=O)C(F)(F)[C@@H]2O
x0195	CN1CCCc2ccc(S(N)(=O)=O)cc21

Table 1 SMILES strings describing the studied compounds

3 Further analysis of QUB-00006-Int-01(R) adaptive sampling simulations

We used the binary-version of nearest-neighbor heavy-atom distance contact between the ligand and MPro residues as feature

[a] QUBIT Pharmaceutical, Incubateur Paris Biotech Santé, 24 rue du Faubourg Saint Jacques 75014 Paris, France; Contact email: davide@qubit-pharmaceuticals.com (DS)
[b] Current affiliation: Chiesi farmaceutici S.p.A, Nuovo centro ricerche, Largo belloli 11a, 43122, Parma (Italy)
[c] Current affiliation: Pharmacelera, Torre R, 4a planta, Despatx A05, Parc Cientific de Barcelona, Baldiri Reixac 8, 08028 Barcelona, (Spain)
[d] University of Applied Sciences and Arts Northwestern Switzerland - School of Life-Sciences, Hofackerstrasse 30, CH-4132 Muttenz, Switzerland
[e] Department of Pharmaceutical and Pharmacological Sciences, University of Padova, via Marzolo 5, 35131, Padova (Italy)
[f] Department of Chemistry, University of Padova, via Marzolo 1, 35131, Padova (Italy)
[g] Molecular Modeling Section, Department of Pharmaceutical and Pharmacological Sciences, University of Padua, via F. Marzolo 5, 35131, Padova, (Italy)
[h] Department of Molecular Medicine, University of Padua, via Gabelli 63, 35121, Padova, (Italy)
[i] Enamine LTD, 78 Chervonotkats'ka str., Kyiv 02094 (Ukraine)
[l] Laboratoire GBCM, EA7528, Conservatoire National des Arts et Métiers, Hesam Université, 2 Rue Conte, 75003 Paris, France
[m] University of Texas at Austin, Department of Biomedical Engineering, TX 78705, USA
[n] Department of Chemistry, Washington University in Saint Louis, MO 63130, USA
[o] Sorbonne Université, Laboratoire de Chimie Théorique, UMR 7616 CNRS, 75005, Paris,France;Contact email: jean-philip.piquemal@sorbonne-universite.fr (JPP)
[p] Institut Universitaire de France, 75005, Paris,France.
‡ These authors contributed equally to this work

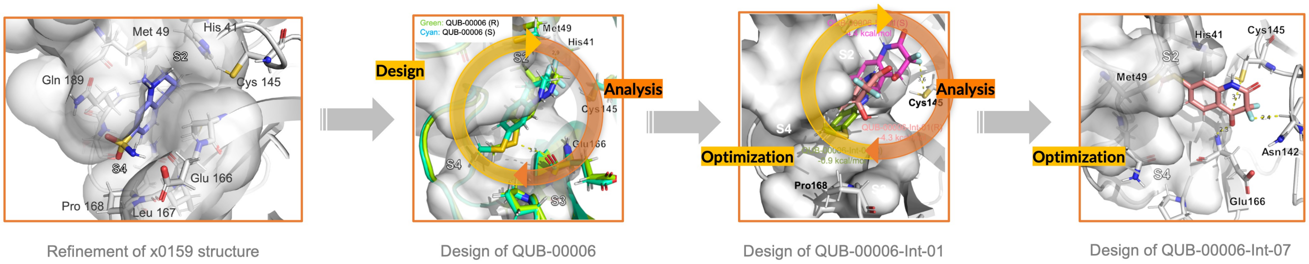

Fig. 1 Schematic summary of our design strategy. First, we select a fragment with an available co-crystal structure with the protein. Then, to increase the binding to Mpro's subpockets, we design new-compounds by introducing chemical modifications to the known binder. Adaptive sampling simulations, free energy simulations, and NMR experiments were used to characterize the binding modes of the newly designed ligands. The promising binding modes were used as a starting point to design covalent ligands.

vector. In this version, if the nearest-neighbor heavy-atom distance between residues is smaller than a threshold, here set to 4 Å, the contact is set 1, otherwise 0. This results in a contact binary feature vector associate with each trajectory frame, reducing the dimension to 608. The 4Å threshold of the feature vector has been chosen in order to find the best compromise between locality and contact information. From then we performed another dimensionality reduction to retain only 10 slow collective variables (CVs): time-lagged variational autoencoder (TICA).TICA approximates the slow CVs by a linear combination of input coordinates. It has been shown in Pérez-Hernández *et al.* [1] that TICA finds the optimal approximation of slow CVs within the class of linear methods. For TICA, we chose a number of dimensions in order to cover 95% of the kinetic variance and scaled them. After optimization, we set the TICA lag-time to 3 ns and retained 12 dimensions. We then used the k-means clustering method on this reduced space. The k-means parameters were fixed to: 200 clusters, 200 maximum number of iteration and a tolerance factor of 10^{-12}. Based on these clusters, we build a Hidden Markov State Model for which we tried different lag times. Here, we find converged values for lag times between 25ns and 50ns and we picked 40ns. After all these steps, we found 3 states to be relevant because of the relatively narrow conformational diversity and the not so pronounced time-scale gaps. For each of the 3 clusters, we randomly extracted 100 structures. To analyze the clusters we focused on the residues which are in contact (within 4Å threshold) with DS7(R) for > 20% of the samples. We computed the mean and standard deviation of: 1) the heavy-atom distance between the ligand and a residue 2) residues RMSD and 3) total RMSD as is presented in the next figure. Although, such AS simulations cannot be considered as fully converged (see reference Jaffrelot Inizan *et al.* [2] for a detailed discussion), they represent a powerful interpretative tools able to locate the potential binding modes.

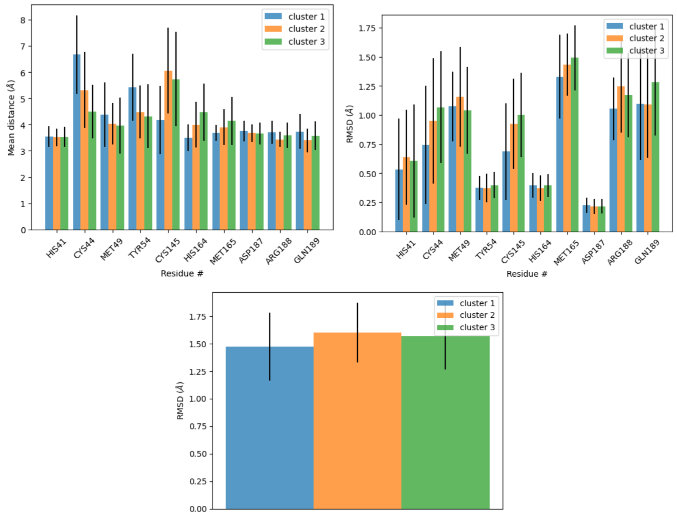

Fig. 2 TICA clusters analysis: a) Heavy-atom distance distribution between drug and residues, b) residues RMSD distribution and c) total RMSD distribution.

4 Experimental details

4.1 Compounds synthesis

Step 1 Synthesis of 2-amino-4-fluorobenzaldehyde A mixture of (2-amino-4-fluorophenyl)methanol (14.2 g, 100.61 mmol) and manganese(IV) oxide (52.48 g, 603.64 mmol) in THF (500 mL) was stirred several days (TLC control) at reflux. The solids were filtrated off and the filtrate was concentrated under reduced pressure to afford 2-amino-4-fluorobenzaldehyde (13.5 g, 97.03 mmol, 96.4% yield).

Step 2 Synthesis of 2-amino-4-(methylsulfanyl)benzaldehyde To an ice-cooled solution of 2-amino-4-fluorobenzaldehyde (16.0 g, 115.0 mmol) in DMSO (50 mL) sodium methylsulfanide (16.12 g, 230.01 mmol, 76.77 ml, 2.0 equiv) was added dropwise. Upon completion of the reaction, the mixture was allowed to warm up to ambient temperature and stirred overnight. The solution was diluted with water and extracted with EtOAc(3x50mL). The organic layer was washed with water, brine, dried over sodium sulfate, and evaporated under reduced pressure to give 2-amino-4-(methylsulfanyl)benzaldehyde (13.0 g, 77.74 mmol, 67.6% yield).

Step 3 Synthesis of 3,3-difluoro-4-hydroxy-7-(methylsulfanyl)-1,2,3,4-tetrahydroquinolin-2-one, QUB-00006Int01 To the mixture of activated zinc (3.6 g, 55.01 mmol) in dry THF, ethyl 2-bromo-2,2-difluoroacetate (9.71 g, 47.84 mmol) was added and the mixture was stirred for 1 hour at room temperature.

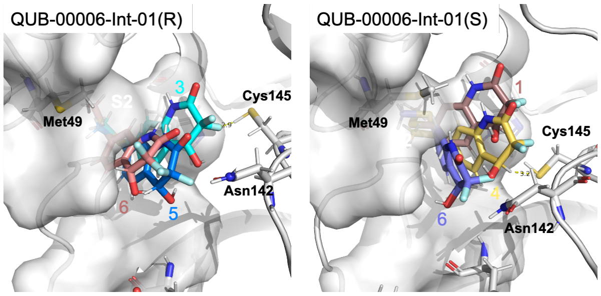

Fig. 3 Exploration of the binding mode of Qub-00006-Int-01 (R) and Qub-00006-Int-01 (S). A) Representative poses of the three clusters of Qub-00006-Int-01 (R) identified during MD simulations performed using our adaptive sampling approach. The 3 clusters represented by poses 3, 5, and 6 are consistent with the respective experimental binding mode of Qub-00006-Int-01 characterized by NMR (STD). Poses 3, 5, and 6 have close binding free energies since the calculative relative binding free energies show a maximum difference of 0.3 kcal/mol between the 3 pairs of clusters (Table 3). B) Representative poses of the three clusters of Qub-00006-Int-01 (S) identified during MD simulations performed using adaptive sampling. The 3 clusters represented by poses 1, 4, and 6 are consistent with the experimental binding mode of Qub-00006-Int-01 characterized by NMR(STD) and have close binding free energies with a difference of less than 0.2 kcal/mol between the 3 pairs of clusters.

Then, 2-amino-4-(methylsulfanyl)benzaldehyde (4.0 g, 23.92 mmol) in THF was added dropwise and the reaction mixture was stirred for a further 19 h at room temperature. The mixture was diluted with ethyl acetate (25x2 mL) and was washed with water (50x2 mL) and brine (50x2 mL), was dried over sodium sulfate, and concentrated under reduced pressure to give pure 3,3-difluoro-4-hydroxy-7-(methylsulfanyl)-1,2,3,4-tetrahydroquinolin-2-one, QUB-00006Int01. Yield: 1000 mg, 17%; Appearance: Orange solid; 1H NMR (500 MHz, DMSO-d6) 10.93 (s, 1H), 7.34 (d, J = 8.1 Hz, 1H), 7.02 – 6.89 (m, 1H), 6.82 (s, 1H), 6.47 (d, J = 5.7 Hz, 1H), 5.00 – 4.84 (m, 1H), 2.44 (s, 3H); HPLC purity: 100%; LCMS: 246.0[M+H]+.

Step 4 Synthesis of 3,3-difluoro-7-(methylsulfanyl)-1,2,3,4-tetrahydroquinoline-2,4-dione, QUB-00006Int07. A mixture of 3,3-difluoro-4-hydroxy-7-(methylsulfanyl)-1,2,3,4-tetrahydroquinolin-2-one (1.3 g, 5.3 mmol), manganese(IV) oxide (4.61 g, 53.0 mmol) and THF (50 mL) was stirred several days (TLC control) at reflux. The solids were filtrated out and the filtrate was concentrated to afford the 3,3-difluoro-7-(methylsulfanyl)-1,2,3,4-tetrahydroquinoline-2,4-dione, QUB-00006Int07. Yield: 1000 mg, 77.6%; Appearance: Yellow solid; 1H NMR (400 MHz, DMSO-d6) 11.32 (s, 1H), 7.74 (d, J = 8.3 Hz, 1H), 7.06 (dd, J = 8.4, 1.8 Hz, 1H), 6.93 (d, J = 1.8 Hz, 1H), 2.54 (s, 3H); HPLC purity: 100%; LCMS: 244.0[M+H]+.

Step 5 Synthesis of 3,3-difluoro-4-hydroxy-4-methyl-7-(methylsulfanyl)-1,2,3,4-tetrahydroquinolin-2-one, QUB-00006Int09. Chloro(methyl)magnesium (322.8 mg, 4.32 mmol, 1.47 ml, 3.0 equiv) was added dropwise to a solution of 3,3-difluoro-7-(methylsulfanyl)-1,2,3,4-tetrahydroquinoline-2,4-dione (350.0 mg, 1.44 mmol) in 30 mL of THF at -70 oC under argon and reaction mixture was stirred at this temperature 1 hour. Then it was quenched with 30mL of saturated aqueous NH4Cl solution and concentrated under vacuum. The residue was partitioned between 50 mL of water and 100 mL of EtOAc. The organic layer was washed with 20 mL of water, brine, dried over sodium sulfate, and concentrated under vacuum to give 3,3-difluoro-4-hydroxy-4-methyl-7-(methylsulfanyl)-1,2,3,4-tetrahydroquinolin-2-one, QUB-00006Int09. Yield: 350 mg, 86.3%; Appearance: Yellow solid; 1H NMR (500 MHz, Chloroform-d) 8.58 (s, 1H), 7.47 (d, J = 8.2 Hz, 1H), 7.01 (dd, J = 8.1, 1.8 Hz, 1H), 6.75 (d, J = 1.9 Hz, 1H), 2.47 (s, 3H), 1.60 (s, 3H); HPLC purity: 100%; LCMS: 260.2[M+H]+.

Step 6 Synthesis of 3,3-difluoro-4-methyl-7-(methylsulfanyl)-1,2,3,4-tetrahydroquinolin-2-one, QUB-00006Int10 3,3-Difluoro-4-hydroxy-4-methyl-7-(methylsulfanyl)-1,2,3,4-tetrahydroquinolin-2-one (308.01 mg, 1.19 mmol) was dissolved in DCM, the mixture was cooled to 0 oC and 2,2,2-trifluoroacetic acid (677.28 mg, 5.94 mmol, 460.0 μl, 5.0 equiv) and triethylsilane (691.6 mg, 5.95 mmol, 950.0 μl, 5.0 equiv) was added. The mixture was heated to 40 oC and stirred 3h at that temperature. After that mixture was cooled to ambient temperature and was washed with water and aq. NaHCO3 solution to give pure 3,3-difluoro-4-methyl-7-(methylsulfanyl)-1,2,3,4-tetrahydroquinolin-2-one, QUB-00006Int10. Yield: 200 mg, 69.2%; Appearance: Beige solid; 1H NMR (600 MHz, DMSO-d6) 10.98 (s, 1H), 7.24 (d, J = 8.0 Hz, 1H), 6.96 (dd, J = 8.0, 1.9 Hz, 1H), 6.84 (d, J = 1.9 Hz, 1H), 3.71 – 3.50 (m, 1H), 2.43 (s, 3H), 1.24 (d, J = 7.1 Hz, 3H); HPLC purity: 100%; LCMS: 244.0[M+H]+.

Step 7 Synthesis of 3,3-difluoro-4-methyl-7-(methylsulfanyl)-1,2,3,4-tetrahydroquinoline, QUB-00006Int08 To a solution of 3,3-difluoro-4-methyl-7-(methylsulfanyl)-1,2,3,4-tetrahydroquinolin-2-one (100.0 mg, 411.06 μmol) in 30 mL of dry THF borane dimethyl sulfide complex (93.73 mg, 1.23 mmol) was added in one portion. The resulting mixture was stirred at 45 oC overnight, then poured into cold K2CO3 aq. solution and extracted with EtOAc. The organic layer was washed with water, brine, dried over sodium sulfate, and evaporated under reduced pressure. The reside was subjected to HPLC to give 3,3-difluoro-4-methyl-7-(methylsulfanyl)-1,2,3,4-tetrahydroquinoline, QUB-00006Int08. Yield: 8.5 mg, 8.6%; Appearance: Yellow oil; 1H NMR (400 MHz, DMSO-d6) 7.15 (d, J = 8.0 Hz, 1H), 6.75 (dd, J = 8.1, 2.0 Hz, 1H), 6.70 (d, J = 2.0 Hz, 1H), 3.76 – 3.62 (m, 1H), 3.62 – 3.53 (m, 1H), 3.39 – 3.23 (m, 1H), 2.55 (s, 3H), 1.51 (d, J = 7.0 Hz, 3H); HPLC purity: 100%; LCMS: 230.2[M+H]+.

The synthesis of 3,3-difluoro-7-(methylsulfanyl)-1,2,3,4-tetrahydroquinolin-4-ol, QUB-00006Int02: to a solution of 3,3-difluoro-4-hydroxy-7-(methylsulfanyl)-1,2,3,4-tetrahydroquinolin-2-one (100.0 mg, 407.75 μmol) in 30 mL of dry THF borane dimethyl sulfide complex (92.93 mg, 1.22 mmol) was added in one portion. The resulting mixture was stirred at 45 oC overnight, then poured into cooled K2CO3 aqua solution and extracted with EtOAc. The organic layer was washed with water, brine, dried over sodium sulfate, and evaporated under reduced pressure. The resulting residue was subjected to HPLC purification to give 3,3-difluoro-7-(methylsulfanyl)-1,2,3,4-tetrahydroquinolin-4-ol, QUB-00006Int02. Yield: 48.9 mg, 51.9%; Appearance: Beige solid; 1H NMR (400 MHz,

Methanol-d4) 7.12 (d, J = 8.0 Hz, 1H), 6.57 (dd, J = 8.0, 1.8 Hz, 1H), 6.52 (d, J = 1.8 Hz, 1H), 4.56 (t, J = 7.6, 6.7 Hz, 1H), 3.69 – 3.51 (m, 1H), 3.42 – 3.34 (m, 1H), 2.42 (s, 3H); HPLC purity: 100%; LCMS: 232.0[M+H]+.

Table 2 List of inactive compounds

No.	ID	MW (g/mol)	Structure
5	Compound 6	232.25	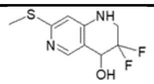
8	Compound 9	246.24	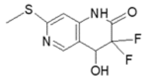
9	Compound 10	263.24	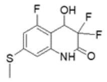
10	Compound 11	231.27	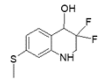
11	Compound 13	264.25	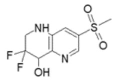
12	Compound 14	248.25	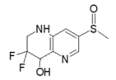
13	Compound 15	184.62	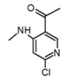
15	Compound 19	190.03	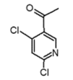
16	Compound 20	263.26	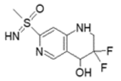
17	Compound 21	323.37	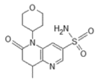
18	Compound 23	196.27	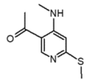
21	Compound 26	232.25	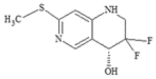
22	Compound 27	232.25	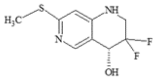
23	Compound 28	206.26	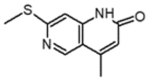
24	Compound 29	243.27	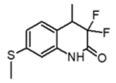
25	Compound 30	259.27	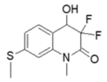
26	Compound 31	259.27	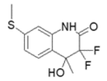
27	Compound 32	231.26	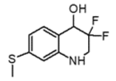
28	Compound 33	246.23	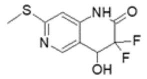
29	Compound 34	277.24	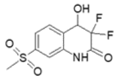

Table 2 continued

No.	ID	MW (g/mol)	Structure
30	Compound 35	274.29	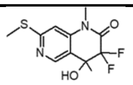
34	Compound 37	217.17	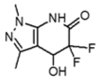
35	Compound 38	274.24	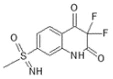
36	Compound 39	257.26	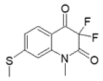

References

1 G. Pérez-Hernández, F. Paul, T. Giorgino, G. De Fabritiis and F. Noé, *The Journal of chemical physics*, 2013, **139**, 07B604_1.

2 T. Jaffrelot Inizan, F. Célerse, O. Adjoua, D. El Ahdab, L.-H. Jolly, C. Liu, P. Ren, M. Montes, N. Lagarde, L. Lagardère, P. Monmarché and J.-P. Piquemal, *Chem. Sci.*, 2021, **12**, 4889–4907.
